# Assessment of Breast Cancer Surgery in Manitoba: A Descriptive Study

**DOI:** 10.3390/curroncol28010058

**Published:** 2021-01-19

**Authors:** Iresha Ratnayake, Pamela Hebbard, Allison Feely, Natalie Biswanger, Kathleen Decker

**Affiliations:** 1 Department of Epidemiology & Cancer Registry, CancerCare Manitoba, Winnipeg, MB R3E 0V9, Canada; afeely@cancercare.mb.ca (A.F.); kdecker@cancercare.mb.ca (K.D.); 2 Department of Community Health Sciences, Rady Faculty of Health Sciences, University of Manitoba, Winnipeg, MB R3E 3P5, Canada; nbiswanger@cancercare.mb.ca; 3 Department of Surgery, Rady Faculty of Health Sciences, University of Manitoba, Winnipeg, MB R3E 3P5, Canada; phebbard@cancercare.mb.ca; 4 CancerCare Manitoba, Winnipeg, MB R3E 0V9, Canada; 5 Screening Programs, CancerCare Manitoba, Winnipeg, MB R3C 2B1, Canada; 6 Research Institute in Oncology & Hematology, CancerCare Manitoba, Winnipeg, MB R3E 0V9, Canada

**Keywords:** breast cancer, breast neoplasms, invasive carcinoma, surgical variation, surgical quality

## Abstract

Background: Variation in breast cancer surgical practice patterns can lead to poor clinical outcomes. It is important to measure and reduce variation to ensure all women diagnosed with breast cancer receive equitable, high-quality care. A population-based assessment of the variation in breast cancer surgery treatment and quality has never been conducted in Manitoba. The objective of this study was to assess the variation in surgical treatment patterns, quality of care, and post-operative outcomes for women diagnosed with invasive breast cancer. Methods: This descriptive study used data from the Manitoba Cancer Registry, Hospital Discharge Abstracts Database, Medical Claims, Manitoba Health Insurance Registry, and Statistics Canada. The study included women in Manitoba aged 20+ and diagnosed with invasive breast cancer between 1 January 2010 and 31 December 2014. Results: Axillary lymph node dissection (ALND) for node-negative disease ranged from 11.8% to 33.3%, timeliness (surgery within 30 days of consult) ranged from 33.3% to 60.2%, and re-excision ranged from 14.7% to 24.6% between health authorities. Women who underwent breast-conserving surgery had the shortest median length of stay and women who underwent mastectomy with immediate reconstruction had the longest median length of stay. In-hospital post-operative complications were higher among women who received mastectomy with immediate reconstruction (9.9%). Conclusion: Variation in surgical treatment, quality, and outcomes exist in Manitoba. The findings from this study can be used to inform cancer service delivery planning, quality improvement efforts, and policy development. Influencing data-driven change at the health system level is paramount to ensuring Manitobans receive the highest quality of care.

## 1. Introduction

Health care quality measurement has evolved into a routine part of health care planning and delivery. Quality is defined as “the degree to which health services for individuals and populations increase the likelihood of desired health outcomes and are consistent with current professional knowledge” [1]. High-quality care is effective, accessible, safe, patient-centered, equitable, and has the capacity to deliver appropriate services [2]. High-quality care leads to improved patient outcomes and better value for money [3]. However, providing high-quality care can be challenging due to growth in technology and new evidence. For example, research expenditure in Canada has increased steadily ($20 billion in 2000 to $34 billion in 2018) along with the number of randomized controlled trials (RCTs) worldwide (2756 RCTs in 2010 to 40,675 in 2019), which has led to a rapid generation of health care knowledge [4,5]. Unfortunately, health care systems can be slow to change and may not adopt new evidence in a timely manner. This may result in variation in practice and quality leading to suboptimal outcomes.

Variations in healthcare practice patterns have been studied for decades among multiple specialties [6,7]. Studies that have examined variation in breast cancer surgery found that treatment varies by institution [8,9], surgeon [9,10], and geographic location [11,12]. For example, the mastectomy rate in Canada ranged from 35% in Manitoba to 61% in Newfoundland and Labrador [13]. Variation in breast cancer surgery also exists within provinces with Ontario reporting that 42% of women living in the Erie St. Clair health region received a mastectomy with lymph node excision compared to 15% of women in Mississauga Halton health region [14]. Variation in breast cancer surgical quality has also been seen with regards to primary tumor management (e.g., positive margin rates) and axillary management (e.g., number of sentinel lymph nodes identified) by the surgeon and by the institution [15]. The reasons for the variation are multi-factorial and may include factors such as diagnostic practices, patient preference, patient health status, technology, local training programs, and financial incentives [16]. As a result of this variation, some patients can experience longer hospital stays, more readmissions, further treatments, increased outpatient visits, and permanent disability, leading to a poor patient experience and increased cost to the healthcare system [5,17,18]. Clearly, it is important to measure and reduce variation in surgery to ensure all women diagnosed with breast cancer receive equitable, high-quality care. In Manitoba, a population-based assessment of the variation in breast cancer surgery treatment and quality has never been conducted. The objective of this study was to assess the variation in surgical treatment patterns, quality of care, and post-operative outcomes for women diagnosed with invasive breast cancer.

## 2. Experimental Section

### 2.1. Study Design and Data Sources

A descriptive study design was used to address the objective stated above. The following data sources were used: Manitoba Cancer Registry (MCR), Hospital Discharge Abstracts Database, Medical Claims Database, Manitoba Health Insurance Registry, and Statistics Canada 2006 Census. The MCR is a population-based registry that is legally mandated to collect, classify, and maintain accurate, comprehensive information about cancer cases including diagnosis date, tumor type and location, stage, and treatment in the province of Manitoba. The MCR was used to identify women diagnosed with invasive breast cancer and to identify surgical procedures. The Medical Claims Database, maintained by Manitoba Health, Seniors and Active Living (MHSAL), is generated by claims filed by health care providers for reimbursement of services and includes services provided, diagnosis, provider, and service date and was used to identify surgical consult dates. The Hospital Discharge Abstracts Database includes all hospital admissions for Manitoba residents and was used to identify surgical procedures, in-hospital complications, and length of stay. The Manitoba Health Insurance Registry contains individual and family-level information including demographic, vital status, and migration information. The Manitoba Health Insurance Registry was used to refine the cohort by ensuring all individuals were eligible for health care coverage and lived in the province during the study period. The 2006 Census data contains information about area-level average household income based on each individual’s area of residence and was used to stratify results by income quintile. All Manitoba residents have been assigned a personal health identification number which was used to link the provincial health information databases. 

### 2.2. Study Population

The study included women in Manitoba aged 20 years or older who were diagnosed with invasive breast cancer (International Classification of Diseases for Oncology (ICD-O) C50.0–50.9 between 1 January 2010 and 31 December 2014. Atypical and rare morphologies including lymphomas and sarcomas of the breast, phyllodes tumors, Paget’s disease, and benign breast lesions were excluded. Manitoba is a Canadian province with publicly funded health care and five regional health authorities (Winnipeg Regional Health Authority, Prairie Mountain Health, Interlake-Eastern Regional Health Authority, Southern Health-Santé Sud, Northern Regional Health Authority). 

Winnipeg Regional Health Authority (WRHA) is the largest urban health authority which includes the City of Winnipeg with a population density of 1518.8 per km^2^ and the northern town of Churchill (16.7 per km^2^) [19]. The overall population density for the WRHA is 1112.3 per km^2^. The second-largest city in Manitoba is Brandon with a population density of 631.2 per km^2^, which is part of Prairie Mountain Health whose overall population density is 2.6 per km^2^. The remaining health authorities have a population density <10 per km^2^ with the majority of northern towns with <1 per km^2^. For example, in the Northern Regional Health Authority, a population of 74,000 people is spread out over 396,000 km^2^ resulting in a population density of 0.18 per km^2^ [19,20]. It is important to acknowledge these differences in geography and population density as it impacts access to services and patient care pathways. For the purposes of this study, we will refer to WRHA as the urban regional health authority (RHA) and the remaining health authorities as the rural regional health authorities (RHAs). 

### 2.3. Definition of Surgical Treatment 

Surgery for the treatment of breast cancer was identified using the following Canadian Classification of Health Interventions (CCI) codes: breast-conserving surgery (1.YM.87, 1.YM.88), mastectomy (1.YM.89, 1.YM.91), mastectomy with immediate reconstruction (1.YM.90, 1.YM.92), and lymph node dissection (1.MD.87, 1.MD.89). All surgical treatments provided within 12 months after a woman’s diagnosis date were included up until 2015. Axillary dissection was defined using the following CCI codes: 1. MD.89, 1.YM.91, and 1.YM.92.

### 2.4. Outcomes

Surgical treatment patterns in Manitoba were identified by calculating the percentage of women who underwent surgery by age group, income quintile, type of surgery, health authority of residence, health authority of surgery, and stage. A literature review was conducted to identify indicators associated with high-quality breast cancer treatment. Final indicators were determined by an expert review led by surgical oncologists specializing in breast cancer treatment. Quality of care was measured using the following indicators: the percentage of women who underwent surgery within 30 days of surgical consult, the percentage of women who underwent a re-excision, and the percentage of women with axillary lymph node dissection for pathologically node-negative disease. In this study, patients who received neoadjuvant chemotherapy were excluded and those who had an axillary dissection following a sentinel lymph node biopsy were not considered to have a negative axillary dissection for the purposes of this analysis. Patients were also excluded if they had stage IV disease. Post-operative outcomes were determined by measuring in-hospital post-operative complications and length of stay.

### 2.5. Statistical Analysis

Descriptive statistics in the form of percentages and 95% confidence intervals (CI) were analyzed using SAS version 9.4. Analyses were stratified by age group (20–49, 50–59, 60–69, 70–79, 80+), income quintile (Urban: U1 (lowest) to U5 (highest) and Rural: R1 (lowest) to R5 (highest)), RHA of residence at time of diagnosis (Winnipeg Regional Health Authority, Prairie Mountain Health, Interlake-Eastern Regional Health Authority, Southern Health-Santé Sud, Northern Regional Health Authority), and stage at diagnosis (stages I to IV). 

## 3. Results

From 2010 to 2014, 3962 women in Manitoba were diagnosed with invasive breast cancer. The age-standardized incidence rate was 165 per 100,000. The median age was 63 (interquartile range [IQR] = 53–73). Among the women who underwent surgery, the majority were under age 70 (69.8%) (Table 1). In both urban and rural settings, women who lived in areas with higher income quintiles had a higher percentage of surgery. The majority of women who underwent surgery lived in the urban RHA (regional health authority) (60.3%) and were diagnosed at stage l or ll (84.3%).

### 3.1. Surgical Treatment Patterns

Surgical treatment patterns in Manitoba are described in Table 2. Among women diagnosed with invasive breast cancer, 92.3% (*n* = 3658) underwent surgical resection. Breast-conserving surgery was the most common procedure (66.7%) followed by mastectomy without immediate reconstruction (22.8%) and mastectomy with immediate reconstruction (10.5%). The youngest age group (20–39) underwent the highest percentage of mastectomy with immediate reconstruction (36.1%; 95% CI: 27.9 to 44.3). This percentage decreased with increasing age. Women in the lowest income quintiles had a lower percentage of mastectomy with immediate reconstruction (6.5% in urban; 95% CI: 4.1 to 8.9; 8.0% in rural; 95% CI: 4.3 to 11.6) compared to women in the highest income quintile (13.9% in urban; 95% CI: 10.8 to 17.0; 13.7% in rural; 95% CI: 9.6 to 17.7). 

**Figure 1:**
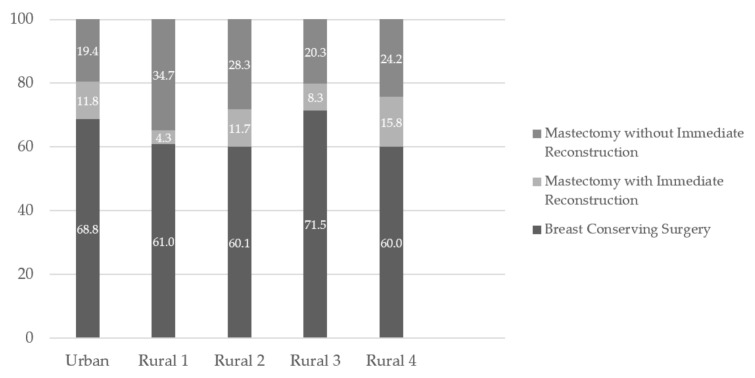
Type of resection for breast cancer, by Regional Health Authority, 2010–2015.

Procedures differed by RHA of residence at diagnosis (Figure 1). Mastectomy with immediate reconstruction was performed for 11.8% (95% CI: 10.6 to 13.2) of women who lived in the urban RHA and ranged from 4.3% (95% CI: 2.6 to 6.1) to 15.8% (95% CI: 8.5 to 23.1) among the rural RHAs. Mastectomy without immediate reconstruction was 19.4% (95% CI: 17.8 to 21.1) for women who lived in the urban RHA and ranged from 20.3% (95% CI: 16.3 to 24.2) to 34.7% (95% CI: 30.6 to 38.8) in the rural RHAs. The percentage of breast-conserving surgery was 68.8% (95% CI: 66.8 to 70.7) in the urban RHA and ranged from 60.0% (95% CI: 50.1 to 69.9) to 71.5% (95% CI: 67.1 to 75.9) in the rural RHAs. Mastectomy with immediate reconstruction showed greater disparities between RHAs with rural RHAs ranging from 4.3% (95% CI: 2.6 to 6.1) to 15.8% (95% CI: 8.5 to 23.1) and the urban RHA showing 11.8% (95% CI: 10.5 to 13.2). The percentage of mastectomy without immediate reconstruction was consistently higher among rural RHAs (20.3% 95% CI: 16.3 to 24.2 to 34.7% 95% CI: 30.6 to 38.8) and 19.4% (95% CI: 17.8 to 21.1) in the urban RHA. Breast-conserving surgery was highest among women with stage I breast cancer (79.0%; 95% CI: 77.1 to 80.9). Contrary to this, mastectomy was highest among women with stage III and IV breast cancer though the absolute numbers of stage IV patients receiving any surgery were quite small (only 67 patients out of 3658 who received surgery). 

### 3.2. Quality of Care 

The quality indicators measured are summarized in Table 3. In Manitoba, 19.6% of women with confirmed node-negative disease received an axillary lymph node dissection. The percentage of women who received ALND for node-negative disease increased with age (2.6% 95% CI: 0.0 to 7.7 in 20–29 versus 29.3% 95% CI: 19.4 to 39.1 in 80+). The percentage of women who underwent ALND for node-negative disease also varied by RHA of residence at diagnosis. Among women who lived in urban RHA, only 11.8% (95% CI: 8.5 to 15.2) underwent this procedure compared to a range of 21.0% (95% CI: 10.8 to 31.1) to 33.3% (95% CI: 26.1 to 40.6) in rural RHAs. Of those patients who received an axillary dissection for node-negative disease, most had stage I cancer. Among those who had surgery in urban RHA, 13.5% (95% CI: 10.6 to 16.4) underwent ALND for node-negative disease compared to 38.0% (95% CI: 29.8 to 46.1) in rural 1 and 42.4% (95% CI: 25.6–59.3) in rural 2.

Fifty percent of women in Manitoba received surgery within 30 days of the first surgical consult. This indicator ranged from 33.3% (95% CI: 20.8 to 45.9) to 60.2% (95% CI: 54.3 to 66.0) depending on the RHA of residence. Among women who underwent breast-conserving surgery, 18.5% underwent a re-excision. Among women who underwent treatment in the urban RHA, 17.5% (95% CI: 15.9 to 19.1) underwent re-excision and this ranged from 23.0% (95% CI: 17.7 to 28.2) to 46.8% (95% CI: 29.6 to 64.2) in rural RHAs. The percentage of women who underwent re-excision after breast-conserving surgery also increased with stage from 15.5% (95% CI: 13.6 to 17.4) for women diagnosed with stage I breast cancer to 32.7% (95% CI: 25.7 to 39.8) for women diagnosed with stage III cancer. 

### 3.3. Post-Operative Outcomes 

The post-operative outcome measures are summarized in Table 4. Women who underwent breast-conserving surgery had the shortest median length of stay (0 days = day surgery) and women who underwent mastectomy with immediate reconstruction had the longest median length of stay (3 days). The percentage of in-hospital post-operative complications were higher among women who received mastectomy with immediate reconstruction (9.9% 95% CI: 7.1 to 12.7) compared to women who had breast-conserving surgery (1.5% 95% CI: 1.1 to 1.9) or those who had a mastectomy without reconstruction (4.6% 95% CI: 3.2 to 5.9).

## 4. Discussion

We found that surgical treatment patterns, quality of care, and post-operative outcomes for women diagnosed with invasive breast cancer from 2010 to 2014 in Manitoba varied by age, income quintile, regional health authority, and stage. The differences that are the most clinically significant are disparities in access to immediate reconstruction and variations in ALND for node-negative disease. With a high five-year survival rate of 88% [21], survivorship and longer-term quality of life issues are important aspects of breast cancer treatment. Mastectomy with immediate reconstruction offers a variety of benefits such as eliminating the need for a second major surgery solely for reconstruction, better cosmetic outcomes, and improved psychological well-being and quality of life [22,23,24]. In Manitoba, 33.3% of women who underwent surgical resection for breast cancer had a mastectomy, which is lower than other jurisdictions such as New Brunswick (47.0%), Nova Scotia (56.0%), and Saskatchewan (65.0%) [13]. Among the women who underwent a mastectomy, 31.4% received a mastectomy with immediate reconstruction which is higher than provinces such as Nova Scotia (3.8%) and Ontario (7.7%) [25,26]. 

The percentage of women who underwent immediate reconstruction varied by RHA and disparities exist within Manitoba. Immediate reconstruction is only available in the city of Winnipeg in the urban RHA and patient pathways possibly contribute to the differences shown in the data. Specialists practicing outside the region must refer patients to this region for the procedure. The two regions with the highest percentage of reconstruction were the urban and northern (rural 4) RHAs. Due to the remote nature of the northern RHA, women most often travel to the urban RHA for treatment. Since patients have to travel to the only facility offering immediate reconstruction for their primary treatment, they are more likely to also opt-in to receive immediate reconstruction. In contrast to this, rural RHA 1 is the only other region (besides urban RHA) to offer surgery, systemic, and radiation therapy locally to their patients. As a result, women living in this region could potentially be choosing non-reconstructive surgery in their home center, where they can also receive the remainder of their care. It may also be possible that providers are more reticent to refer patients to another site, for a variety of reasons. Thus, patient and provider preferences may play a role in the variation shown in the data. However, as per the pan-Canadian standards for breast cancer surgery [27]. all patients undergoing mastectomy should be informed of their reconstructive options and this interchange should be documented in the patient chart. The differences in mastectomy with immediate reconstruction are statistically significant between the urban RHA and rural 1 RHA. Other studies have reported similar findings regarding geographical variation in reconstructive surgery [28]. Histology characteristics did not differ by RHA, X^2^ (8, N = 3658) = 4.29, *p* > 0.05 (Table S1). In order to further understand the underlying reasons behind the disparities in immediate reconstruction, future studies should incorporate chart reviews and/or qualitative research questions. 

In addition to disparities between regions, disparities between income quintiles were also identified with statistically significant differences between the lowest and highest income quintiles. Histology characteristics did not differ by income quintile, X^2^ (18, N = 3559) = 9.56, *p* > 0.05 (Table S2). Women living in areas with the lowest income quintile had the lowest percentage of mastectomy with immediate reconstruction. This finding is similar to other studies that have reported immediate reconstruction to be lower among individuals with lower socioeconomic status [29,30]. This could be due to systemic barriers with regards to access or personal choices such as not wanting to take a longer absence from work due to longer recovery time associated with reconstruction and/or a lack of available sick leave. Women also have the choice of undergoing delayed reconstruction following curative treatment. While not measured in the current study, wait times for delayed reconstruction in Manitoba are several years long [31]. Therefore, the variation identified in this study is more concerning as patients who do not have access to immediate reconstruction are also less likely to receive delayed reconstruction within an acceptable timeframe.

Sentinel lymph node biopsy (SLNB) is the standard of care in breast cancer patients who are clinically node-negative [32]. The latest 2016 guideline by the American Society of Clinical Oncology states that “clinicians should not recommend axillary lymph node dissection for women with early-stage breast cancer who do not have nodal metastases” [33]. This recommendation is based on high-quality evidence and the strength of the recommendation is strong. Axillary lymph node dissection (ALND) is less favorable due to dramatically increased risk of lymphedema and lower quality of life compared to SLNB [32]. The proportion of patients with node-negative disease who do not undergo axillary clearance is a key quality indicator regarding overtreatment identified by the European Society of Breast Cancer Specialists (EUSOMA) [34]. The minimum standard for this indicator is 80% and the target is 90%. Initiatives such as Choosing Wisely Canada have attempted to increase awareness about this topic in recent years [35]. In our study, we found that 19.6% of women in Manitoba with node-negative cancer underwent an ALND; other studies from other jurisdictions found this number to be as high as 49% [36]. Therefore, Manitoba meets the minimum standard published by EUSOMA but not the target. We also found a large degree of variability in the percentage of women who had ALND across the province. Statistically significant differences exist between urban RHA and rural RHAs 1 and 2. The European Society of Breast Cancer Specialists (EUSOMA) has identified that a lack of multidisciplinary breast centers leads to challenges regarding unnecessary axillary dissections [37]. Our calculation excludes patients who underwent neoadjuvant chemotherapy as these patients are more likely to undergo ALND for clinically acceptable reasons. Therefore, the variation identified is not due to higher occurrences of ALND in this group. A large body of evidence exists regarding volume and outcome relationships in breast cancer surgery [37]. Higher surgeon and hospital volume are associated with improved outcomes, and further research should use this lens to understand the root causes behind these disparities.

Strengths of this study include the use of population-based data which permitted geographic comparisons, limited missing data, the use of administrative databases that have been evaluated for completeness, reliability, and validity, and the development of indicators based on a comprehensive review of the literature. However, several limitations should be noted. Income is an area-level measure that was used as a proxy for individual-level income. This may result in some misclassification of an individual’s actual income. However, several studies have shown a substantial correlation between area-level and self-reported individual-level income [38,39]. Post-operative complication data are often not captured on the discharge abstracts; therefore, some complications may be missing. Data about patient choice were unavailable but would provide meaningful insight into understanding variation if incorporated into future research. 

## 5. Conclusions

In conclusion, we have identified variations in practice patterns, quality of care, and post-operative outcomes for breast cancer surgery in Manitoba. Further research is needed to determine the reasons for the identified variations. Quality improvement initiatives, such as further training and audit and feedback reports, can be implemented to reduce variation and improve the quality of care and are being actively pursued by team members.

## Figures and Tables

**Table 1:** Characteristics of women diagnosed with invasive breast cancer, Manitoba, 2010–2014.

Characteristic	Total	Had Surgery N (%)	Did not Have Surgery N (%)
**Manitoba**	3962 (100.0)	3658 (92.3)	304 (7.7)
**Age Group**			
20–49	664 (16.8)	631 (17.2)	33 (10.9)
50–59	919 (23.2)	880 (24.1)	39 (12.8)
60–69	1091 (27.5)	1042 (28.5)	49 (16.1)
70–79	752 (19.0)	705 (19.3)	47 (15.5)
80+	536 (13.5)	400 (10.9)	136 (44.7)
**Income Quintile**			
Urban 1 (lowest)	450 (11.7)	401 (17.4)	49 (26.9)
U2	491 (12.8)	457 (19.9)	34 (18.7)
U3	531 (13.8)	497 (21.6)	34 (18.7)
U4	511 (13.3)	479 (20.8)	32 (17.6)
U5 (highest)	501 (13.0)	468 (20.3)	33 (18.1)
Rural 1 (lowest)	238 (6.2)	213 (16.9)	25 (25.0)
R2	276 (7.2)	254 (20.2)	22 (22.0)
R3	275 (7.2)	260 (20.7)	15 (15.0)
R4	272 (7.1)	252 (20.0)	20 (20.0)
R5 (highest)	296 (7.7)	278 (22.1)	18 (18.0)
**RHA of Residence (at diagnosis)**			
Urban	* (60.6)	2206 (60.3)	* (64.5)
Rural 1	* (13.8)	511 (14.0)	* (11.2)
Rural 2	* (12.2)	446 (12.2)	* (12.2)
Rural 3	* (10.9)	400 (10.9)	* (10.8)
Rural 4	* (2.5)	95 (2.6)	* (1.3)
**Stage**			
Stage l	1764 (44.9)	1699 (46.6)	65 (23.0)
Stage ll	1447 (36.8)	1374 (37.7)	73 (25.9)
Stage lll	545 (13.9)	506 (13.9)	39 (13.8)
Stage lV	172 (4.4)	67 (1.8)	105 (37.2)

* Suppressed due to cell sizes *n* < 5.

**Table 2:** Surgical treatment patterns among women who underwent resection for invasive breast cancer, Manitoba, 2010–2015.

Characteristic	Breast-Conserving Surgery		Mastectomy with Immediate Reconstruction		Mastectomy without Immediate Reconstruction	
	N	% (95% CI)	N	% (95% CI)	N	% (95% CI)
**Total**	2439	66.7	383	10.5	836	22.8
**Age Group**						
20–39	56	42.1 (33.7, 50.5)	48	36.1 (27.9, 44.3)	29	21.8 (14.8, 28.8)
40–49	291	58.4 (54.1, 62.8)	128	25.7 (21.9, 29.5)	79	15.9 (12.7, 19.1)
50–59	590	67.0 (63.9, 70.2)	135	15.3 (13.0, 17.7)	155	17.6 (15.1, 20.1)
60–69	755	72.5 (69.7, 75.2)	66	6.3 (4.9, 7.8)	221	21.2 (18.7, 23.7)
70–80+	747	67.6 (64.8, 70.4)	6	0.5 (0.1, 1.0)	352	31.9 (29.1, 34.6)
**Income Quintile**						
Urban 1 (lowest)	277	69.1 (64.6, 73.6)	26	6.5 (4.1, 8.9)	98	24.4 (20.2, 28.6)
U2	299	65.4 (61.1, 69.8)	43	9.4 (6.7, 12.1)	115	25.2 (21.2, 29.1)
U3	355	71.4 (67.5, 75.4)	48	9.7 (7.1, 12.3)	94	18.9 (15.5, 22.4)
U4	325	67.8 (63.7, 72.0)	70	14.6 (11.5, 17.8)	84	17.5 (14.1, 20.9)
U5 (highest)	331	70.7 (66.6, 74.8)	65	13.9 (10.8, 17.0)	72	15.4 (12.1, 18.7)
Rural 1 (lowest)	129	60.6 (54.0, 67.1)	17	8.0 (4.3, 11.6)	67	31.5 (25.2, 37.7)
R2	157	61.8 (55.8, 67.8)	15	5.9 (3.0, 8.8)	82	32.3 (26.5, 38.0)
R3	162	62.5 (56.7, 68.4)	15	5.8 (2.9, 8.6)	82	31.7 (26.0, 37.3)
R4	151	59.9 (53.9, 66.0)	35	13.9 (9.6, 18.2)	66	26.2 (20.8, 31.6)
R5 (highest)	191	68.7 (63.3, 74.2)	38	13.7 (9.6, 17.7)	49	17.6 (13.1, 22.1)
**RHA of Residence (at diagnosis)**						
Urban	1517	68.8 (66.8, 70.7)	261	11.8 (10.5, 13.2)	428	19.4 (17.8, 21.1)
Rural 1	311	61.0 (56.7, 65.2)	22	4.3 (2.6, 6.1)	177	34.7 (30.6, 38.8)
Rural 2	268	60.1 (55.5, 64.6)	52	11.7 (8.7, 14.6)	126	28.3 (24.1, 32.4)
Rural 3	286	71.5 (67.1, 75.9)	33	8.3 (5.6, 10.9)	81	20.3 (16.3, 24.2)
Rural 4	57	60.0 (50.1, 69.9)	15	15.8 (8.5, 23.1)	23	24.2 (15.6, 32.8)
**RHA of Surgery**						
Urban RHA	2148	67.5 (65.9, 69.9)	383	12.0 (10.9, 13.2)	652	20.5 (19.1, 21.9)
Rural 1	248	64.9 (60.1, 69.7)	n/a		134	35.1 (30.3, 29.9)
Rural 2–4	32	43.8 (32.5, 55.2)	n/a		41	56.2 (44.8, 67.5)
Out of Province	11	57.9 (35.7, 80.1)	n/a		8	42.1 (19.9, 64.3)
**Stage**						
Stage l	1342	79.0 (77.1, 80.9)	113	6.7 (5.5, 7.8)	244	14.4 (12.7, 16.0)
Stage ll	889	64.7 (62.2, 67.3)	146	10.6 (9.0, 12.3)	338	24.6 (22.3, 26.9)
Stage lll	171	33.8 (29.7, 37.9)	111	21.9 (18.3, 25.5)	224	44.3 (39.9, 48.6)
Stage lV	28	41.8 (30.0, 53.6)	12	17.9 (8.7, 27.1)	27	40.3 (28.6, 52.0)

**Table 3:** Surgical quality among women who underwent surgical resection for invasive breast cancer, Manitoba, 2010–2015.

Characteristic	Axillary Lymph Node Dissection for Node Negative Disease		≤30 Days between First Surgical Consult and First Surgery		Re-Excision after Breast-Conserving Surgery	
	N	% (95% CI)	N	% (95% CI)	N	% (95% CI)
**Manitoba**	137	19.6	1, 245	49.3	450	18.5
**Age Group**						
20–39	-	2.6 (0.0, 7.7)	27	32.9 (22.8, 43.1)	20	35.7 (23.2, 48.3)
40–49	-	13.2 (7.0, 19.4)	176	49.4 (44.2, 54.6)	70	24.1 (19.1, 29.0)
50–59	-	12.1 (7.1, 17.1)	299	46.7 (42.9, 50.6)	118	20.0 (16.8, 23.2)
60–69	-	25.0 (18.4, 31.6)	409	54.0 (50.4, 57.5)	133	17.6 (14.9, 20.3)
70–79	-	26.3 (18.9, 33.6)	227	48.6 (44.1, 53.1)	76	15.7 (12.5, 19.0)
80+	-	29.3 (19.4, 39.1)	107	48.0 (41.4, 54.5)	33	12.5 (8.5, 16.5)
**RHA of Residence (at diagnosis)**						
Urban	-	11.8 (8.5, 15.2)	766	47.9 (45.5, 50.4)	258	17.0 (15.1, 18.9)
Rural 1	-	33.3 (26.1, 40.6)	160	60.2 (54.3, 66.0)	73	23.5 (18.8, 28.2)
Rural 2	-	24.0 (15.4, 32.5)	151	49.2 (43.6, 54.8)	66	24.6 (19.5, 29.8)
Rural 3	-	21.0 (10.8, 31.1)	150	49.8 (44.2, 55.5)	42	14.7 (10.6, 18.8)
Rural 4	-	23.5 (3.4, 43.7)	18	33.3 (20.8, 45.9)	11	19.3 (9.1, 29.5)
**RHA of Surgery**						
Urban 1	71	13.5 (10.6, 16.4)	n/a		376	17.5 (15.9, 19.1)
Rural 1	52	38.0 (29.8, 46.1)	n/a		57	23.0 (17.7, 28.2)
Rural 2	14	42.4 (25.6, 59.3)				
Rural 2–4	n/a	n/a	n/a	n/a	15	46.8 (29.6, 64.2)
**Stage**						
Stage l	80	88.9 (82.4, 95.4)	609	49.6 (46.8, 52.4)	208	15.5 (13.6, 17.4)
Stage ll	57	18.6 (14.3, 23.0)	496	52.2 (49.0, 55.4)	179	20.1 (17.5, 22.8)
Stage lll	0	0.0 (0.0, 0.0)	140	40.1 (35.0, 45.3)	56	32.7 (25.7, 39.8)

**Table 4:** Post-operative outcomes among women who underwent surgical resection for invasive breast cancer, Manitoba, 2010–2015.

Length of Stay	Median (Days)	90th Percentile (Days)
Breast-Conserving Surgery	0	1
Mastectomy without Immediate Reconstruction	1	4
Mastectomy with Immediate Reconstruction	3	5
**In-Hospital Complication**	**N**	**% (95% CI)**
Breast-Conserving Surgery	43	1.5 (1.1, 1.9)
Mastectomy without Immediate Reconstruction	42	4.6 (3.2, 5.9)
Mastectomy with Immediate Reconstruction	43	9.9 (7.1, 12.7)

## Data Availability

Restrictions apply to the availability of these data. Data was obtained from CancerCare Manitoba Health and Seniors Care. These data are available with the permission of CancerCare Manitoba and Manitoba Health and Seniors Care.

## Supplementary Materials

The following are available online at https://www.mdpi.com/1718-7729/28/1/58/s1, Table S1: Histopathological Characteristics among Regional Health Authorities of Residence, Table S2: Histopathological Characteristics among Income Quintiles.
