# Correction: Ratnayake et al. Assessment of Breast Cancer Surgery in Manitoba: A Descriptive Study. *Curr. Oncol.* 2021, *28*, 581–592

**DOI:** 10.3390/curroncol28040242

**Published:** 2021-07-20

**Authors:** Iresha Ratnayake, Pamela Hebbard, Allison Feely, Natalie Biswanger, Kathleen Decker

**Affiliations:** 1 Department of Epidemiology & Cancer Registry, CancerCare Manitoba, Winnipeg, MB R3E 0V9, Canada; afeely@cancercare.mb.ca (A.F.); kdecker@cancercare.mb.ca (K.D.); 2 Department of Community Health Sciences, Rady Faculty of Health Sciences, University of Manitoba, Winnipeg, MB R3E 3P5, Canada; nbiswanger@cancercare.mb.ca; 3 Department of Surgery, Rady Faculty of Health Sciences, University of Manitoba, Winnipeg, MB R3E 3P5, Canada; phebbard@cancercare.mb.ca; 4 CancerCare Manitoba, Winnipeg, MB R3E 0V9, Canada; 5 Screening Programs, CancerCare Manitoba, Winnipeg, MB R3C 2B1, Canada; 6 Research Institute in Oncology & Hematology, CancerCare Manitoba, Winnipeg, MB R3E 0V9, Canada

The authors wish to make a correction to this paper due to a minor change in indicator definition [1]. The overall findings of the study remain unchanged; however, we wish to include the updated data in the manuscript.

In the original article, the abstract stated that the axillary lymph node dissection for node-negative disease ranged from 11.8% to 33.3%. The range should be corrected to 3.4% to 32.6%.

In Section 3.4, the original article stated, “The quality indicators measured are summarized in Table 3. In Manitoba, 19.6% of women with confirmed node-negative disease received an axillary lymph node dissection. The percentage of women who received ALND for node-negative disease increased with age (2.6% 95% CI: 0.0 to 7.7 in 20–29 vs. 29.3% 95% CI: 19.4 to 39.1 in 80+). The percentage of women who underwent ALND for node-negative disease also varied by RHA of residence at diagnosis. Among women who lived in urban RHA, only 11.8% (95% CI: 8.5 to 15.2) underwent this procedure compared to a range of 21.0% (95% CI: 10.8 to 31.1) to 33.3% (95% CI: 26.1 to 40.6) in rural RHAs. Of those patients who received an axillary dissection for node-negative disease, most had stage I cancer. Among those who had surgery in urban RHA, 13.5% (95% CI: 10.6 to 16.4) underwent ALND for node-negative disease compared to 38.0% (95% CI: 29.8 to 46.1) in rural 1 and 42.4% (95% CI: 25.6–59.3) in rural 2”.

It should be replaced with the following,

“The quality indicators measured are summarized in Table 3. In Manitoba, 19.6% of women who underwent an axillary lymph node dissection were node negative. When looking at the percentage of node negative patients who underwent axillary dissection however, 5.8% of women with confirmed node-negative disease received an axillary lymph node dissection. This number was variable when looking at certain demographic factors. The percentage of women who received ALND for node-negative disease increased with age (1.9% 95% CI: 0.0 to 5.7 in 20–39 versus 7.9% 95% CI: 4.9 to 10.9 in 80+). The percentage of women who underwent ALND for node-negative disease also varied by RHA of residence at diagnosis. Among women who lived in urban RHA, only 3.0% (95% CI: 2.1 to 3.9) underwent this procedure compared to a range of 4.8% (95% CI: 2.3 to 7.4) to 15.9% (95% CI: 12.0 to 19.8) in rural RHAs. Of those patients who received an axillary dissection for node-negative disease, most had stage II cancer. Among those who had surgery in urban RHA, 3.4% (95% CI: 2.6 to 4.2) underwent ALND for node-negative disease compared to 20.4% (95% CI: 15.4 to 25.3) in rural 1 and 32.6% (95% CI: 18.6 to 46.6) in rural 2”.

In the original article, Table 3 was as follows:

**Table 3:** Surgical quality among women who underwent surgical resection for invasive breast cancer, Manitoba, 2010–2015.

Characteristic	Axillary Lymph Node Dissection for Node Negative Disease		≤30 Days Between First Surgical Consult and First Surgery		Re-excision After Breast-Conserving Surgery	
	N	% (95% CI)	N	% (95% CI)	N	% (95% CI)
Manitoba	137	19.6	1245	49.3	450	18.5
**Age Group**						
20–39	-	2.6 (0.0, 7.7)	27	32.9 (22.8, 43.1)	20	35.7 (23.2, 48.3)
40–49	-	13.2 (7.0, 19.4)	176	49.4 (44.2, 54.6)	70	24.1 (19.1, 29.0)
50–59	-	12.1 (7.1, 17.1)	299	46.7 (42.9, 50.6)	118	20.0 (16.8, 23.2)
60–69	-	25.0 (18.4, 31.6)	409	54.0 (50.4, 57.5)	133	17.6 (14.9, 20.3)
70–79	-	26.3 (18.9, 33.6)	227	48.6 (44.1, 53.1)	76	15.7 (12.5, 19.0)
80+	-	29.3 (19.4, 39.1)	107	48.0 (41.4, 54.5)	33	12.5 (8.5, 16.5)
**RHA of Residence (at diagnosis)**						
Urban	-	11.8 (8.5, 15.2)	766	47.9 (45.5, 50.4)	258	17.0 (15.1, 18.9)
Rural 1	-	33.3 (26.1, 40.6)	160	60.2 (54.3, 66.0)	73	23.5 (18.8, 28.2)
Rural 2	-	24.0 (15.4, 32.5)	151	49.2 (43.6, 54.8)	66	24.6 (19.5, 29.8)
Rural 3	-	21.0 (10.8, 31.1)	150	49.8 (44.2, 55.5)	42	14.7 (10.6, 18.8)
Rural 4	-	23.5 (3.4, 43.7)	18	33.3 (20.8, 45.9)	11	19.3 (9.1, 29.5)
**RHA of Surgery**						
Urban 1	71	13.5 (10.6, 16.4)	n/a		376	17.5 (15.9, 19.1)
Rural 1	52	38.0 (29.8, 46.1)	n/a		57	23.0 (17.7, 28.2)
Rural 2	14	42.4 (25.6, 59.3)				
Rural 2–4	n/a	n/a	n/a	n/a	15	46.8 (29.6, 64.2)
**Stage**						
Stage I	80	88.9 (82.4, 95.4)	609	49.6 (46.8, 52.4)	208	15.5 (13.6, 17.4)
Stage II	57	18.6 (14.3, 23.0)	496	52.2 (49.0, 55.4)	179	20.1 (17.5, 22.8)
Stage III	0	0.0 (0.0, 0.0)	140	40.1 (35.0, 45.3)	56	32.7 (25.7, 39.8)

Table 3 should be replaced with the following:

**Table 3:** Surgical quality among women who underwent surgical resection for invasive breast cancer, Manitoba, 2010–2015.

Characteristic	Axillary Lymph Node Dissection for Node Negative Disease (*n* = 2379)		≤30 Days Between First Surgical Consult and First Surgery (*n* = 2526)		Re-excision After Breast-Conserving Surgery (*n* = 2439)	
	*n*	% (95% CI)	*n*	% (95% CI)	*n*	% (95% CI)
Manitoba	137	5.8	1245	49.3	450	18.5
Age Group						
20–39	-	1.9 (0.0, 5.7)	27	32.9 (22.8, 43.1)	20	35.7 (23.2, 48.3)
40–49	-	6 (3.1, 9.0)	176	49.4 (44.2, 54.6)	70	24.1 (19.1, 29.0)
50–59	-	3.8 (2.2, 5.4)	299	46.7 (42.9, 50.6)	118	20.0 (16.8, 23.2)
60–69	-	5.4 (3.8, 7.0)	409	54.0 (50.4, 57.5)	133	17.6 (14.9, 20.3)
70–79	-	7.3 (5.0, 9.6)	227	48.6 (44.1, 53.1)	76	15.7 (12.5, 19.0)
80+	-	7.9 (4.9, 10.9)	107	48.0 (41.4, 54.5)	33	12.5 (8.5, 16.5)
RHA of Residence (at diagnosis)						
Urban	-	3.0 (2.1, 3.9)	766	47.9 (45.5, 50.4)	258	17.0 (15.1, 18.9)
Rural 1	-	15.9 (12.0, 19.8)	160	60.2 (54.3, 66.0)	73	23.5 (18.8, 28.2)
Rural 2	-	8.0 (4.9, 11.2)	151	49.2 (43.6, 54.8)	66	24.6 (19.5, 29.8)
Rural 3	-	4.8 (2.3, 7.4)	150	49.8 (44.2, 55.5)	42	14.7 (10.6, 18.8)
Rural 4	-	7.1 (0.4, 13.9)	18	33.3 (20.8, 45.9)	11	19.3 (9.1, 29.5)
RHA of Surgery						
Urban 1	71	3.4 (2.6, 4.2)	n/a		376	17.5 (15.9, 19.1)
Rural 1	52	20.4 (15.4, 25.3)	n/a		57	23.0 (17.7, 28.2)
Rural 2	14	32.6 (18.6, 46.6)				
Rural 2–4	n/a	n/a	n/a	n/a	15	46.8 (29.6, 64.2)
Stage						
Stage I	80	4.9 (3.9, 6.0)	609	49.6 (46.8, 52.4)	208	15.5 (13.6, 17.4)
Stage II	57	7.7 (5.8, 9.6)	496	52.2 (49.0, 55.4)	179	20.1 (17.5, 22.8)
Stage III	0	0.0 (0.0, 0.0)	140	40.1 (35.0, 45.3)	56	32.7 (25.7, 39.8)

n/a: The RHA of surgery stratification is not applicable for some indicators.

In the original article, the discussion stated the following, “In our study, we found that 19.6% of women in Manitoba with node-negative cancer underwent an ALND; other studies from other jurisdictions found this number to be as high as 49% [36]. Therefore, Manitoba meets the minimum standard published by EUSOMA but not the target”.

It should be replaced with the following,

“In our study, we found that 5.8% of women in Manitoba with node-negative cancer underwent an ALND, but that the number varied greatly by surgery geographic locations; other studies from other jurisdictions found this number to be as high as 49% [36]. Therefore, Manitoba meets the minimum standard published by EUSOMA as well as the target, but on a global scale, but some centers are falling well behind this benchmark”.

The authors apologize for any inconvenience caused and state that the scientific conclusions are unaffected. The original article has been updated.
