# Supplementary material for: Assessment of Breast Cancer Surgery in Manitoba: A Descriptive Study
Source: Curr Oncol. 2021 Jan 19;28(1):581–92. doi: 10.3390/curroncol28010058 (PMC7903285; doi:10.3390/curroncol28010058)
Supplement: Supplementary file 1 [file curroncol-28-00058-s001.pdf]

Supplementary Materials

Table S1. Histopathological Characteristics among Regional Health Authorities of Residence.

Regional Health Authority of Residence	Ductal Carcinoma N (%)	Lobular Carcinoma N (%)	Other N (%)	Chi-Square (*p*-Value)
Urban	1707 (77.4)	392 (17.8)	107 (4.9)	
Rural 1	379 (74.2)	102 (20.0)	30 (5.9)	
Rural 2	304 (76.0)	77 (19.3)	19 (4.8)	4.29 (0.82)
Rural 3	345 (77.4)	78 (17.5)	23 (5.2)	
Rural 4	77 (81.1)	13 (13.7)	<6 (5.3)	

Table S2. Histopathological Characteristics among Income Quintiles.

Income Quintile	Ductal Carcinoma N (%)	Lobular Carcinoma N (%)	Other N (%)	Chi-Square (*p*-value)
Urban 1 (lowest)	301 (75.1)	78 (19.5)	22 (5.5)	
U2	354 (77.5)	82 (17.9)	21 (4.6)	
U3	385 (77.46)	81 (16.3)	31 (6.2)	
U4	368 (76.8)	91 (19.0)	20 (4.2)	
U5 (highest)	358 (76.5)	87 (18.6)	23 (4.9)	9.56 (0.94)
Rural 1 (lowest)	165 (77.5)	18.8 (18.8)	8 (3.8)	
R2	196 (77.2)	42 (16.5)	16 (6.3)	
R3	197 (75.8)	53 (20.4)	10 (3.9)	
R4	189 (75.0)	46 (18.3)	17 (6.8)	
R5 (highest)	217 (78.1)	49 (17.6)	12 (4.3)	
